# Supplementary material for: Exploiting correlations in multi-coincidence Coulomb explosion patterns for differentiating molecular structures using machine learning
Source: Nat Commun. 2025 Dec 12;16:11366. doi: 10.1038/s41467-025-66369-5 (PMC12727871; doi:10.1038/s41467-025-66369-5)
Supplement: Supplementary file 1 — Supplementary Information [file 41467_2025_66369_MOESM1_ESM.pdf]

Supplementary Information for Exploiting correlations in multi-coincidence Coulomb explosion patterns for differentiating molecular structures using machine learning

Anbu Selvam Venkatachalam, Loren Greenman, Joshua Stallbaumer, Artem Rudenko, Daniel Rolles, and Huynh Van Sa Lam*

James R. Macdonald Laboratory, Kansas State University, Manhattan, KS 66506, USA

(Dated: October 26, 2025)

In this Supplementary Information, we provide additional results and analysis details that are useful to a subset of readers, who are interested in more specialized content, but that are not essential to comprehend the main results of the article.

CONTENTS

Supplementary Note 1. On the possibility of extending the current framework to larger molecular systems 2
- A. Emerging experiment technologies 2
- B. Data analysis pipelines 2

Supplementary Note 2. Additional experimental data 3

Supplementary Note 3. Additional information on the simulation model 5
- A. Initial geometry 5
- B. Comparison between experimental data and Coulomb explosion simulations 5

Supplementary Note 4. Additional discussion on the machine learning analysis 7
- A. Comparing different data reduction techniques 7
- B. On the stochastic nature of UMAP and Random Forest Classifier 8
- C. Machine-learning analysis under varied simulation conditions 9
- D. Data reduction of different channels 12
  - 1. Experimental data 12
  - 2. Simulated data 12
- E. Supporting figures for Random Forest Classification analysis 14

References 16

* huynhlam@ksu.edu

Supplementary Note 1. ON THE POSSIBILITY OF EXTENDING THE CURRENT FRAMEWORK TO LARGER MOLECULAR SYSTEMS

"Complete" CEI is particularly powerful because it delivers, in every single shot, full structural information containing all the atoms in the molecule. However, capturing all ionic fragments from a total molecular breakup in coincidence—and then processing that data—is extremely challenging. The six- and eight-ion coincidences, together with the data analysis framework, demonstrated here, therefore constitute a significant advance over earlier efforts. Nevertheless, we anticipate that extending such high-fold coincidence measurements to larger molecular systems will soon become feasible, driven by experimental advances including higher repetition-rate and intense light sources, improved detector technologies, and more sophisticated analysis approaches.

A. Emerging experiment technologies

Light sources

- Increased repetition rates: Given that a laser of only 3-kHz was used in our work, increasing the repetition rate of the light source will significantly enhance event statistics (e.g., 100-kHz lasers are commercially available, and the LCLS-II XFEL is currently operating at several tens of kHz and will soon be scaled up to 1-MHz operation).
- High-intensity X-ray: Using high-intensity X-ray for ionization will give a much larger population in highly charged states as compared to strong-field ionization (where low charged states are dominant as in this work). X-ray Free Electron Laser with MHz repetition rate, such as LCLS-II, can be an ideal source for this.

Advanced detector technologies: Using even better detectors, such as a HEX anode instead of the QUAD anode that we are using, or a pixel-independent detector (TimePix camera and similar concepts), which further improves the detection efficiency for "identical" particles (with similar m/q). Currently, we can detect up to 4 such identical particles in a molecule. Simply using a bigger detector (e.g., 120 mm compared to the current 80-nm diameter) also helps with detection efficiently; however, the commercial availability might be limited.

Better vacuum: Simply having a better vacuum will allow more light intensity to be used before the data is swamped by background hits. Our vacuum is about 1^{-10} mbar, which can be improved by a factor of 10 with a better pumping scheme.

Such improvements are already underway, exemplified by LCLS's ongoing development of the DREAM Endstation [1] and next-generation detectors [2] optimized for multiparticle coincidence experiments at MHz repetition rates.

B. Data analysis pipelines

Other "complete" CEI channels:

- Exploring options in data analysis, such as analyzing channels where fragments with the same m/q have different charges, will further mitigate the detection efficiency problem since those hits will arrive at the detector at different positions and different times.
- In fact, our data contains many other "complete" CEI fragmentation channels (Fig. 4). How to combine information from all these channels to obtain a better picture of the molecule and its dynamics (with a potential to also increase statistics) is of significant interest [3].

"Incomplete" CEI channels: Applying our method to "incomplete" events—where structural information is partially missing—is an interesting endeavor.

- For events involving incomplete breakup but complete detection, sufficient information remains to distinguish between different dynamical pathways. Our framework can handle these scenarios without modification, though it will lack specific details about the motion of atoms that do not break up.
- For events involving complete ionization but incomplete detection [such as detecting only five atomic fragments for DCE, e.g., $(H^+, C^+, C^+, {}^{35}Cl^+, {}^{35}Cl^+)$], structural information will be incomplete. Specifically, in this example, the information of one hydrogen atom is lost, causing each molecular structure to appear as two distinct clusters because the detected H^+ randomly samples one of two possible hydrogen atoms. This introduces additional complexity in the analysis. Although one might attempt to reconstruct the complete molecular structure

through multivariate analysis [4], this approach would require specific assumptions about molecular geometry and the ionization process.

- In cases where structural dynamics occur predominantly within a localized region—such as intramolecular proton transfer—detecting protons along with two reference markers for the molecular frame could suffice, assuming that the carbon backbone remains largely intact.

All of these scenarios represent intriguing avenues for future research, but they lie beyond the current scope and may be explored in subsequent studies.

Supplementary Note 2. ADDITIONAL EXPERIMENTAL DATA

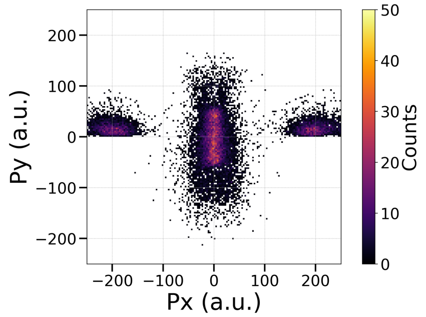

Supplementary Figure 1. **Alternative Newton plot for *trans*-1,2-DCE six-body coincidence channel**. Experimental Coulomb Explosion Imaging (CEI) patterns for *trans*-1,2-DCE, obtained from the six-fold coincidence channel (H^+, H^+, C^+, C^+, $^{35}Cl^+$, $^{35}Cl^+$). The coordinate frame for each event is defined such that the x-axis is set by the vector difference of the two Cl^+ ions, while the xy-plane is established by their vector sum. Due to their back-to-back emission, the momentum sum of the two Cl^+ ions is near zero, leading to an inherently less well-defined xy-reference plane.

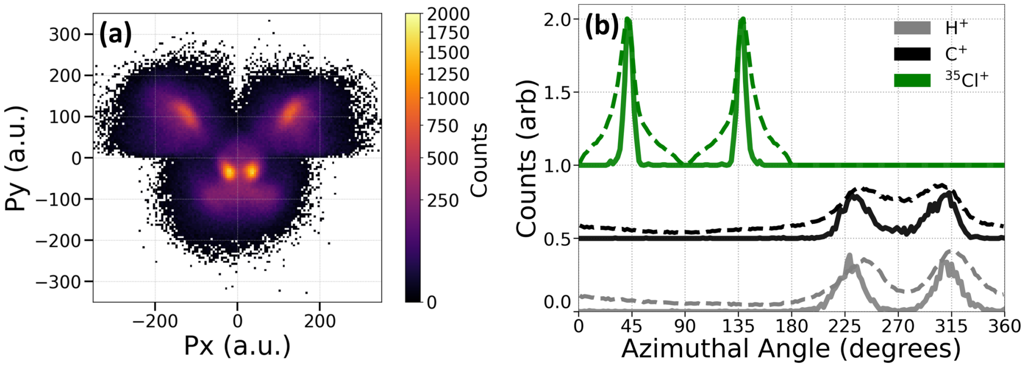

Supplementary Figure 2. **Newton plot for *cis*-1,2-DCE four-fold coincidence channel and distributions of azimuthal angles.** (a) Newton plot for *cis*-1,2-DCE, obtained from the four-body incomplete coincidence channel (H^+, C^+, $^{35}Cl^+$, $^{35}Cl^+$). The reference frame is defined by the vector difference of the two Cl^+ ions along the x-axis, with their sum lying in the upper xy-plane. (b)Yields of H^+, C^+, and $^{35}Cl^+$ fragments as a function of the azimuthal angle in the molecular frame. Solid lines correspond to the six-body complete coincidence fragmentation channel, while dashed lines represent the four-body incomplete coincidence channel involving H^+, C^+, and two $^{35}Cl^+$ ions. Distributions for C^+ and $^{35}Cl^+$ are vertically offset for clarity.

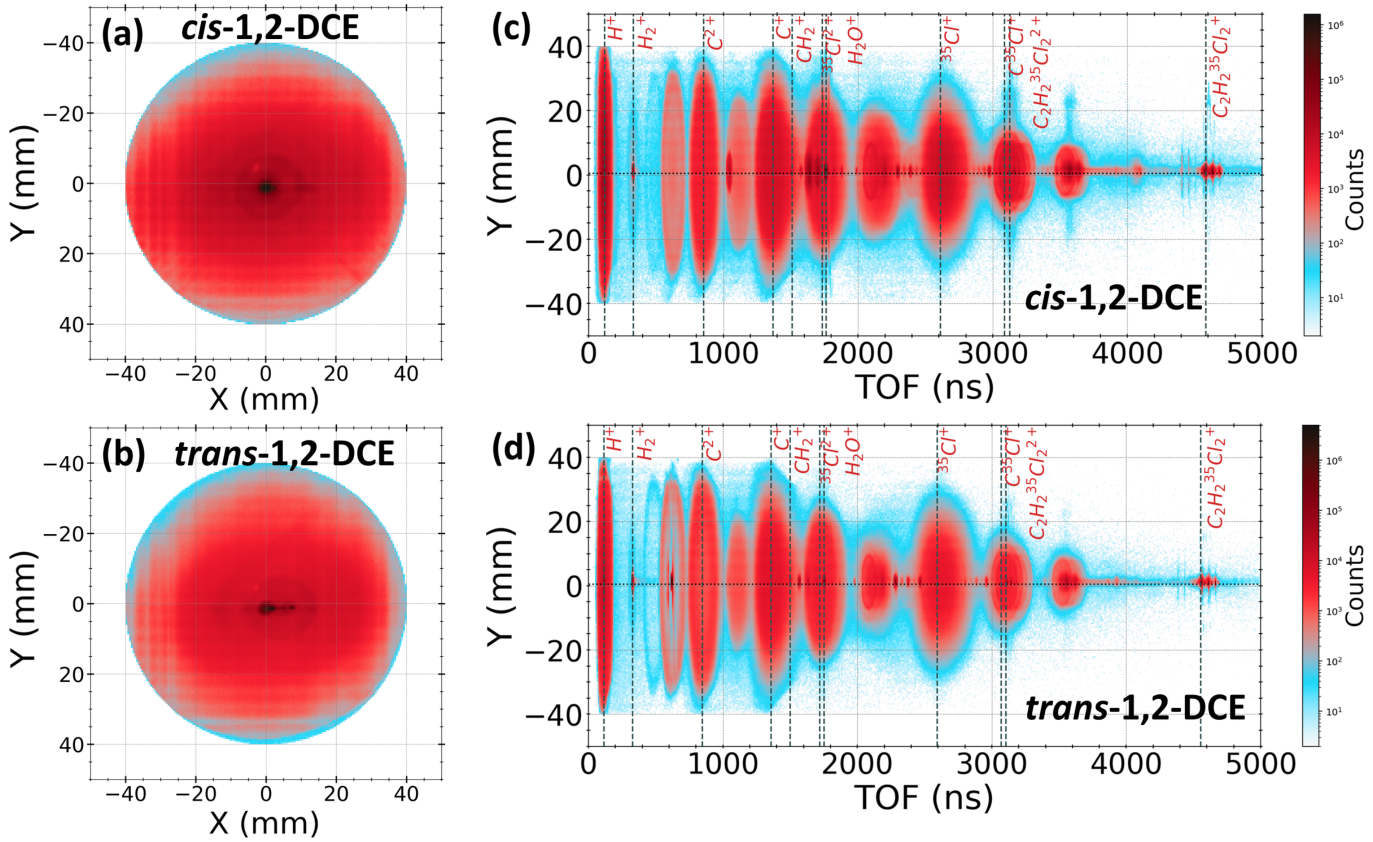

Supplementary Figure 3. **Comparison of the experimental noncoincidence ion imaging data for *cis*- and *trans*-1,2-dichloroethylene (1,2-DCE).** Panels (a) and (b) show the detector-plane XY ion images integrated over all time-of-flight (TOF) values for the *cis*- and *trans*-1,2-DCE isomers, respectively. Panels (c) and (d) display the corresponding Y-TOF maps, i.e., ion yield as a function of TOF and vertical position on the detector, for *cis*- (c) and *trans*-1,2-DCE (d). Vertical dashed lines mark the major fragment ion species, and color represents intensity on a logarithmic scale.

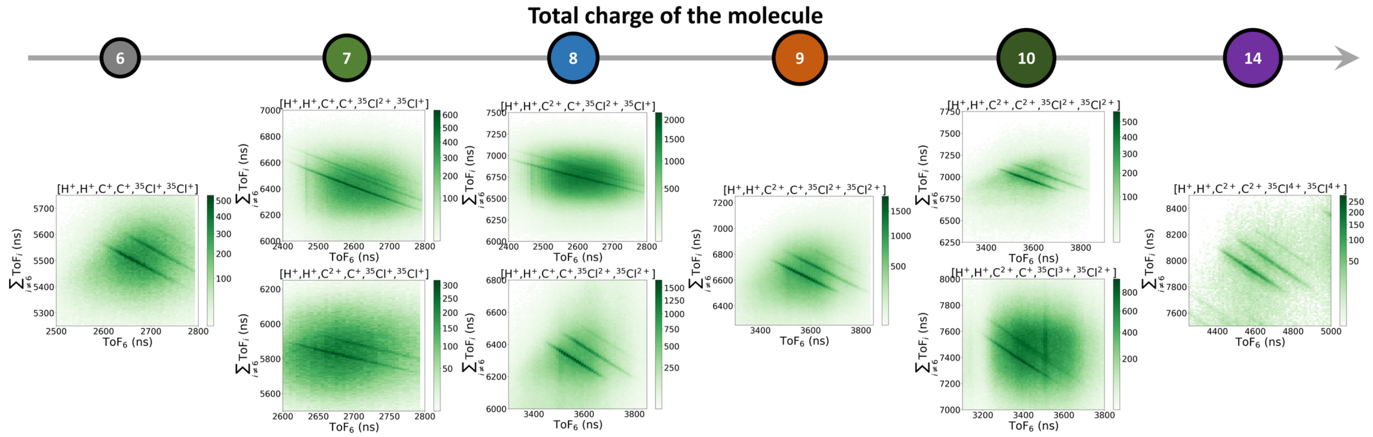

Supplementary Figure 4. **Time-of-flight coincidence maps of exemplary "complete" CEI channels.** The numbers on the top indicate the total charge of the final state. Multiple lines in each panel correspond to channels with different isotopes of Cl ions. Colorbar indicates actual counts of coincident events

Supplementary Note 3. ADDITIONAL INFORMATION ON THE SIMULATION MODEL

A. Initial geometry

Supplementary Table I. Equilibrium geometry of *cis*-1,2-DCE optimized at the B3LYP/aug-cc-pVDZ level.

cis-1,2-DCE				
	Atom	x (Å)	y (Å)	z (Å)
1	C	0.66699	0.96658	0.00000
2	C	-0.66700	0.96658	0.00000
3	H	1.22333	1.90229	0.00000
4	H	-1.22333	1.90229	0.00000
5	Cl	1.66770	-0.45304	0.00000
6	Cl	-1.66770	-0.45305	0.00000

Supplementary Table II. Equilibrium geometry of *trans*-1,2-DCE optimized at the B3LYP/aug-cc-pVDZ level.

trans-1,2-DCE				
	Atom	x (Å)	y (Å)	z (Å)
1	C	-0.36871	0.55424	0.00000
2	C	0.36871	-0.55424	0.00000
3	H	-1.45653	0.56855	0.00000
4	H	1.45653	-0.56855	0.00000
5	Cl	0.36871	2.13823	0.00000
6	Cl	-0.36871	-2.13823	0.00000

Supplementary Table III. Equilibrium geometry of 1,1-DCE optimized at the B3LYP/aug-cc-pVDZ level.

1,1-DCE				
	Atom	x (Å)	y (Å)	z (Å)
1	H	0.93847	2.30932	0.00000
2	H	-0.93847	2.30932	0.00000
3	C	0.00000	1.75934	0.00000
4	C	0.00000	0.42806	0.00000
5	Cl	1.46564	-0.52185	0.00000
6	Cl	-1.46564	-0.52185	0.00000

Supplementary Table IV. *Twisted*-1,2-DCE transient-state geometry between *cis*- and *trans*-1,2-DCE, where the C=C bond rotates 90° from the *cis*-1,2-DCE equilibrium, optimized at the B3LYP/aug-cc-pVDZ level.

twisted-1,2-DCE				
	Atom	x (Å)	y (Å)	z (Å)
1	C	-0.53475	1.19362	-0.31733
2	C	0.26899	0.32851	0.48543
3	H	-1.08439	1.95034	0.26295
4	H	0.15112	-0.00884	1.52174
5	Cl	-1.66406	-0.43127	-0.05612
6	Cl	1.81276	-0.22016	-0.10819

B. Comparison between experimental data and Coulomb explosion simulations

Supplementary Figure 5 shows a comparison between Coulomb explosion simulations using an initial Wigner distribution and the modified initial condition used in the main text in how well they can reproduce the experimental distributions of angles between fragment momentum vectors. Supplementary Figure 6 contrasts measured versus

simulated absolute momentum distributions for H^+, C^+ and Cl^+ ions from the (H^+, H^+, C^+, C^+, Cl^+, Cl^+) channel. The simulation systematically overestimates all fragment momenta magnitudes for both isomers.

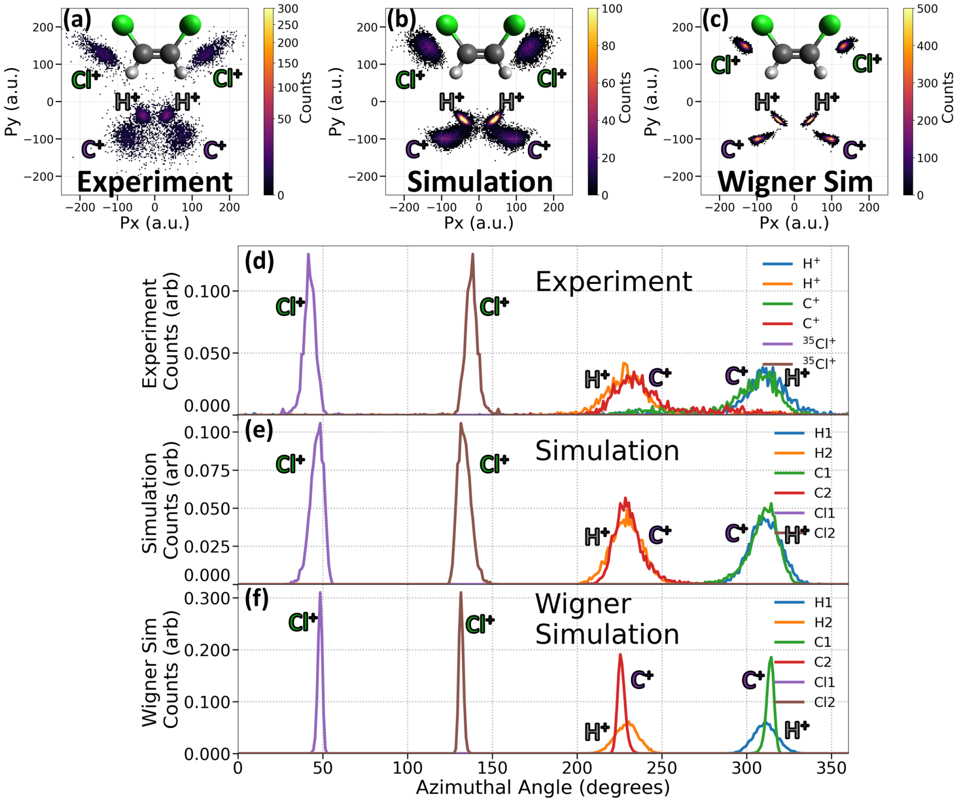

Supplementary Figure 5. **CEI patterns for *cis*-1,2-DCE: experiment, simulation from random geometries, simulation from a Wigner ensemble.** Panels (a), (b) and (c) show the Newton plots of momenta projected onto the p_x–p_y plane. For each event, the coordinate frame is aligned such that the vector difference between the two Cl^+ ions defines the p_x axis, and the vector sum of the two Cl^+ lies in the upper $p_x p_y$ plane. Panels (d), (e) and (f) show the corresponding azimuthal angle distributions, integrated over momentum magnitude, for each ion species. The azimuthal angle is measured counterclockwise from the p_x axis, and normalized to unit area.

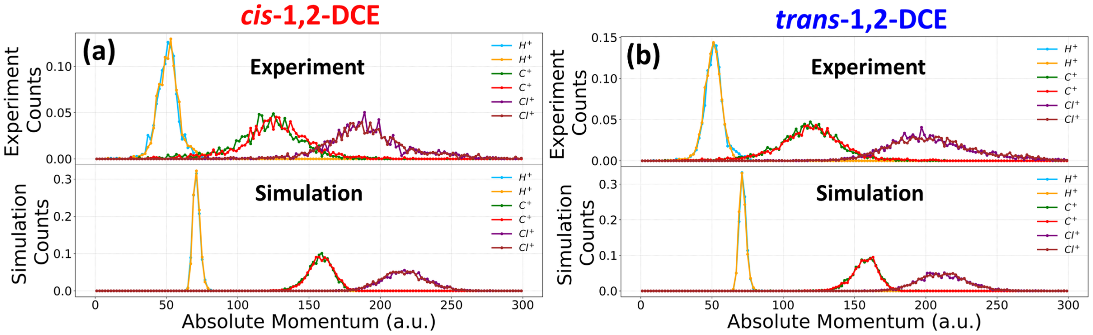

Supplementary Figure 6. **Comparison between the measured (top) and simulated (bottom) absolute momenta of coincident ions from Coulomb explosion imaging of (a) *cis*-1,2-DCE and (b) *trans*-1,2-DCE molecules.** The figures show the distributions for H^+, C^+, and Cl^+ ions. The simulation systematically overestimates the magnitude of the momenta of the fragments.

Supplementary Note 4. ADDITIONAL DISCUSSION ON THE MACHINE LEARNING ANALYSIS

A. Comparing different data reduction techniques

In this section, we provide results from different data reduction techniques, PCA and t-SNE, for a comparison with the UMAP method shown in the main text. Technically, PCA is a linear technique that projects data onto orthogonal axes of maximal variance, preserving global structure but missing nonlinear relationships. t-SNE is a nonlinear, probabilistic method that excels at preserving local neighborhoods but can distort global distances and scales poorly to large datasets [5]. UMAP is a non-linear dimensionality reduction technique designed to preserve both local and some global structures of high-dimensional data while being computationally efficient, built upon the concepts from fuzzy topology and Riemannian geometry [6]. The results of UMAP often look quite similar to those of t-SNE because both use graph layout algorithms to arrange data in a low-dimensional space, but UMAP tends to perform better than t-SNE in the aspects mentioned above. We performed data reduction on two different data sets: one is the experimental data containing two isomers (*cis*- and *trans*-1,2 DCE) [Supplementary Figure 7], and the other is the simulated data containing four different geometries (*cis*-, *trans*-, *twisted*-1,2 DCE and 1,1-DCE) [Supplementary Figure 8].

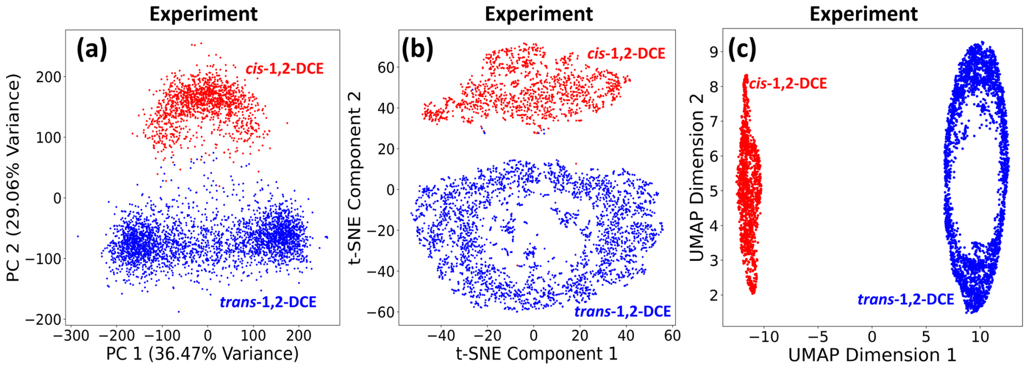

Supplementary Figure 7. Dimensionality reduction of experimental data containing a mixture of *cis* and *trans* geometries. a) Principal Component Analysis (PCA), (b) t-distributed Stochastic Neighbor Embedding (t-SNE) and (c) Uniform Manifold Approximation and Projection (UMAP) dimensionality reduction methods are employed with identical experimental data as inputs. The events are colored by their true labels. In this case, the data is very robust such that all these data reduction and clustering techniques can separate the two isomers in 2D representations.

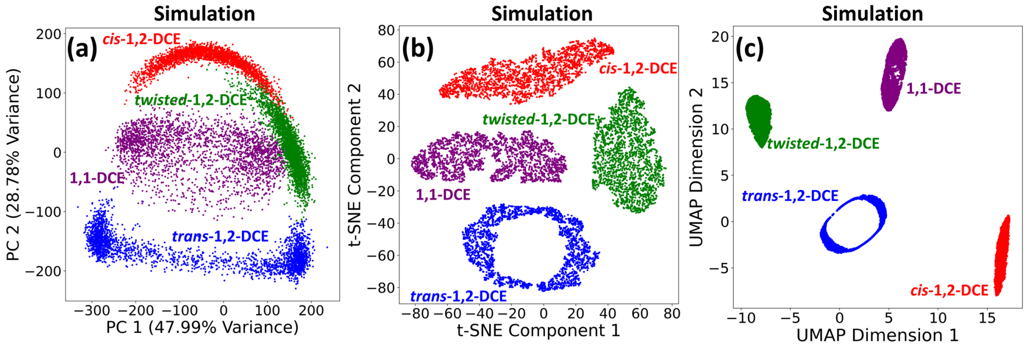

Supplementary Figure 8. Dimensionality reduction of the simulated data containing isomer geometries of 1,2-DCE (*cis, trans, twisted*) and 1,1-DCE. (a) Principal Component Analysis (PCA), (b) t-distributed Stochastic Neighbor Embedding (t-SNE) and (c) Uniform Manifold Approximation and Projection (UMAP) are used with identical simulated data as inputs. The events are colored by their true labels. This result indicates that UMAP and t-SNE are more robust than PCA in separating isomers.

UMAP visually looks better than t-SNE, and t-SNE, in turn, looks better than PCA. To qualitatively quantify the clustering quality of each dimensionality-reduction method, we computed the Silhouette Score [7] and Davies–Bouldin Index [8] for each method as shown below. The Silhouette Score measures how well each point fits within its own cluster versus other clusters, yielding a value between −1 and 1 where higher means clearer, tighter grouping. The

Davies–Bouldin Index evaluates clustering by comparing the average similarity of each cluster to its most similar other cluster, taking values in the range $[0, \infty)$, with lower scores indicating more compact, well-separated clusters. The values are printed in the table below and visualized in Supplementary Figure 9.

We can see that in the case of experimental data containing two isomers, the performance of PCA and t-SNE is roughly similar, while UMAP is distinctly better. Additionally, with simulated data containing four geometries, the performance of PCA drops quickly, while t-SNE is roughly the same as before, and UMAP still yields the best result.

	Experimental data (two isomers)			Simulated data (four isomers)		
Techniques	PCA	t-SNE	UMAP	PCA	t-SNE	UMAP
Silhouette Score	0.466	0.454	0.834	0.295	0.465	0.806
Davies–Bouldin Index	0.881	0.824	0.226	1.457	0.845	0.319

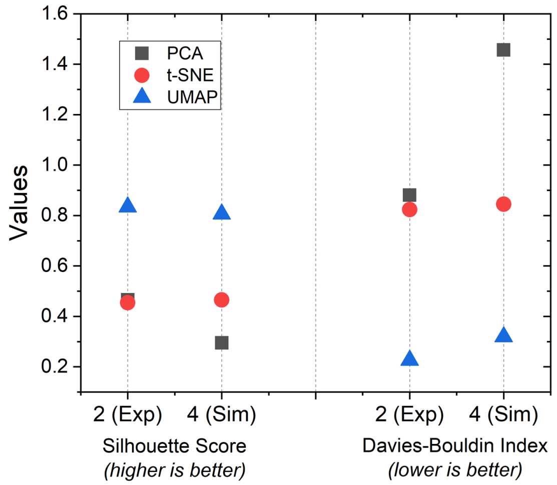

Supplementary Figure 9. Cluster metrics across methods. Silhouette Score and Davies–Bouldin Index calculated for different data reduction methods (PCA, t-SNE, UMAP) on experimental data containing two isomers and simulated data containing four different geometries.

In general, UMAP performs better for clustering because its nonlinear manifold approximation can uncover complex, curved structures and cluster separations (similar to t-SNE) that PCA's straight-line projections cannot. It does this by constructing high- and low-dimensional fuzzy simplicial complexes and minimizing their cross-entropy, thereby capturing both fine-grained local connectivity and broader global trends rather than just maximizing linear variance. This is of particular importance, as shown in the next section, for supervised machine learning where simulated data can be used to guide experimental analysis. For large data sets with complicated structures such as proteins, the same conclusion was reached while comparing PCA and nonlinear techniques such as t-SNE or UMAP [9, 10]. In practice, the actual performance depends on the specific case. In cases where the data can be separated by linear projections, PCA is preferred due to its computational efficiency, simplicity, and interpretability.

B. On the stochastic nature of UMAP and Random Forest Classifier

UMAP inherently includes a stochastic component: the "random state" hyperparameter. This parameter initializes the random number generator, which causes the UMAP result to vary between repeated runs on the same dataset. To evaluate the stability of our UMAP analysis, we repeated the dimensionality reduction procedure 100 times, each time initializing the algorithm with a different randomly assigned "random state", and calculated the Silhouette Score [7] and Davies–Bouldin Index [8]. The resulting metrics (see table below) indicate that our UMAP analysis is highly stable and robust.

	UMAP
Silhouette Score	0.84 ± 0.04
Davies-Bouldin Index	0.22 ± 0.05

Similarly, due to the stochastic nature of the Random Forest Classifier—originating from its initialization with a pseudorandom number ("random state")—its results vary slightly with different random states. To address this variability, we repeated the classification analysis multiple times with different random states and reported the mean and standard deviation of the relative discriminative power (as shown in Fig. 4 and Fig. 5 of the main text).

C. Machine-learning analysis under varied simulation conditions

To test the robustness of our structure identification using the dimensionality reduction approach, we examined how different spreads of possible product geometries affect the ability to differentiate between structures by considering the four scenarios listed in Supplementary Table V.

Supplementary Table V. Simulation parameters for different cases.

	**Simulation parameters**			
	Case I	Case II	Case III	Case IV
cis-1,2-DCE	0.25 A, 0.50 eV	0.25 A, 0.50 eV	0.25 Å, 0.50 eV	0.50 Å, 6.00 eV
trans-1,2-DCE	0.25 Å, 0.50 eV	0.25 Å, 0.50 eV	0.25 Å, 0.50 eV	0.50 Å, 6.00 eV
twisted-1,2-DCE	0.25 Å, 0.50 eV	0.50 Å, 3.00 eV	0.50 Å, 6.00 eV	0.50 Å, 6.00 eV
1,1-DCE	0.25 Å, 0.50 eV	0.50 Å, 3.00 eV	0.50 Å, 6.00 eV	0.50 Å, 6.00 eV

Case I, where the spread of all geometries is minimal, mimicking early delay times when most deposited photon energy remains as electronic excitation. Here, UMAP cleanly separates each geometry into its own cluster (Fig. 5), as extensively discussed in the main text. Case II assigns 3 eV of kinetic energy to the *twisted*-1,2-DCE and 1,1-DCE, modeling partial relaxation on the potential-energy surfaces with some energy converted into nuclear motion. In this case, the UMAP 2D projection shows partial overlap between clusters, but simulated data can be used to guide experimental analysis, as also discussed in the main text (Fig. 6).

In this section, we provide two more cases (III and IV). Case III represents complete relaxation back to the ground states (6 eV kinetic energy, corresponding to a 200 nm pump). Its classification performance (Supplementary Figure 10) matches that of Case II, with supervised UMAP + CEI cleanly separating all isomers into distinct clusters. Case IV is an extreme scenario where all geometries are widely spread to challenge the sensitivity of our analysis. As expected, overall accuracy falls (Supplementary Figure 11). However, the majority of *cis* and *trans* isomers can still be identified correctly, and the experimental data of *cis*- and *trans*--1,2-DCE (gray) show good overlap with their respective simulation clusters. Of the four isomers, *trans* displays the most distinct geometry and thus can be classified with high accuracy, whereas *cis*—whose geometry shares more similarity with 1,1-DCE [e.g., $\angle(C^+, C^+)$, $\angle(H^+, H^+)$]—is more challenging to classify.

Finally, for a comparison, we provide results using Principal Component Analysis (PCA) and Linear Discriminant Analysis (LDA) (Supplementary Figure 12). PCA finds orthogonal axes of maximal variance while LDA optimizes axes for maximal separation between the four geometries. Both PCA and LDA only use linear combinations of the original momentum components. PCA and LDA should be compared with unsupervised UMAP and supervised UMAP, respectively. Their projections (Supplementary Figure 12) show heavy overlap between clusters, making the classification of molecular structures much more challenging. This underscores the need for high-fidelity nonlinear mapping (such as UMAP) to classify molecular structures reliably.

It is worth noting that less isomer separability [if they are still somewhat separable as in Supplementary Figure 12(b)] demands more accurate simulations to classify isomers correctly. In contrast, UMAP combined with "complete" CEI preserves both global structures (large-scale changes between different isomers) and local structural nuances (smaller-scale variation of each isomer), reducing demands on the fine-level details in the simulated data. It is also worth noting that we have not yet been able to obtain good classification using "incomplete" channels (with missing atomic ions). Although this is still a topic of investigation, it highlights the important role of "complete" CEI mode, where very rich structural information, including all atoms in the molecules, is encoded. Thus, the unique pairing of "complete" CEI and UMAP delivers reliable structure identification even under severe scatter.

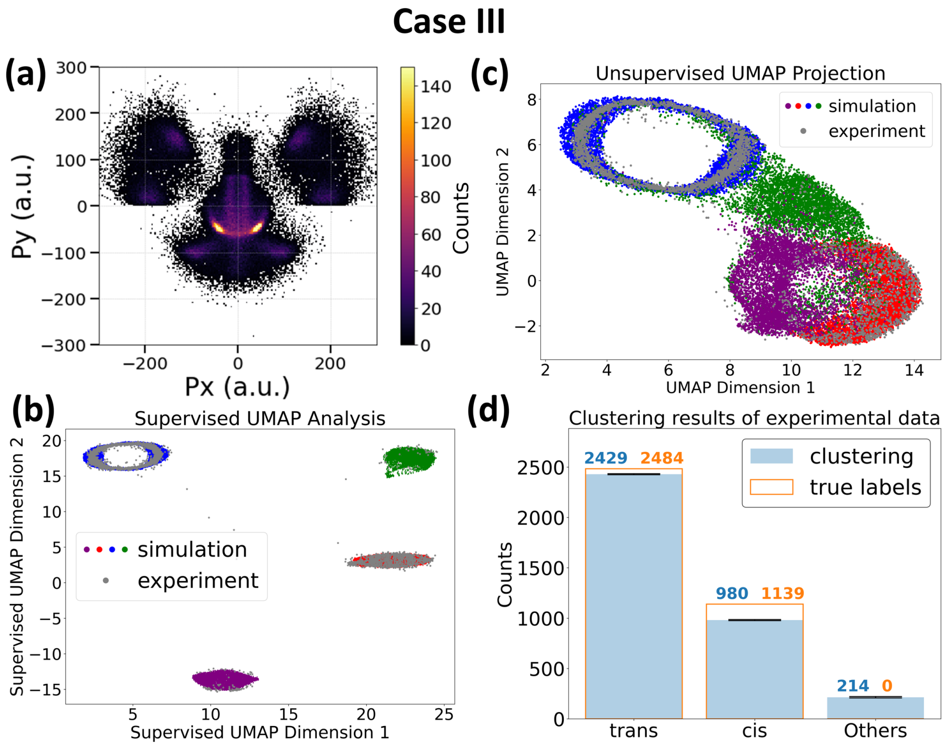

Supplementary Figure 10. **UMAP analysis for complete relaxation to ground state.** (a) Newton map (Px vs Py) from the simulated Case III data. (b) Supervised UMAP embedding trained on the simulation. simulation points are colored by their true isomer labels, and experimental cis/trans events are overlaid in gray. (c) Unsupervised UMAP of the simulated set, colored by true isomer labels with the experimental cis/trans events shown in gray. (d) Experimental clustering summary: filled bars are the cluster assignments, outlined bars are counts by true labels. Error bars show the standard error of the mean across 100 repetitions with different UMAP random states and clustering initializations.

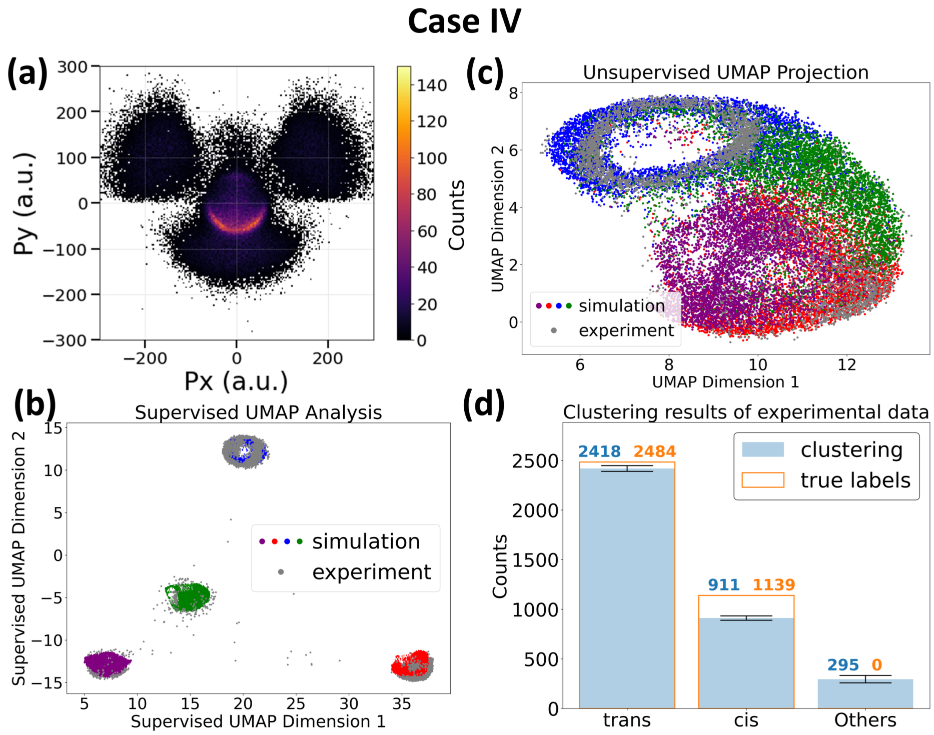

Supplementary Figure 11. UMAP analysis for geometries that are widely spread. (a) Newton map from the Case IV simulation. (b) Supervised UMAP trained on the simulation dataset; simulated points are colored by true isomer, with experimental cis/trans events overlaid in gray. (c) Unsupervised UMAP of the Case IV simulation, again colored by true isomer and the experimental cis/trans events in gray for comparison. (d) Experimental assignment summary: filled bars = clustered counts and the outlined bars = true-label counts. Error bars show standard error across 100 repetitions with different UMAP random states and clustering initializations.

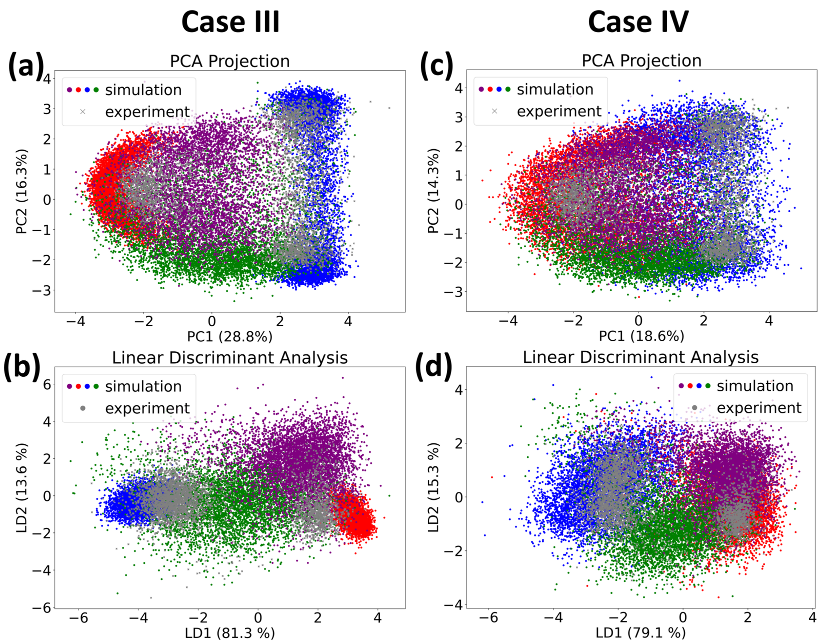

Supplementary Figure 12. PCA and LDA projections for simulated and exprimental data. Panels (a) and (b) show the PCA projections for the simulated datasets for Case III and Case IV, respectively. The LDA of the same datasets is shown in panels (c) and (d), respectively. In all panels, points are colored by true isomer labels, with experimental data overlaid in gray.

D. Data reduction of different channels

In this section, we examine the influence of final charge states on data reduction. The first subsection (Supplementary Figure 13) presents results from experimental data containing *cis*- and *trans*-1,2-DCE isomers. The second subsection (Supplementary Figures 14-15) presents results from simulated data for the four geometries discussed in the main text. For simulated data, both the spatial spread and the kinetic energy imparted to the molecules were varied. Final states with higher total charges provide better separation between geometries. This improvement arises because: (1) the ratio between the width of the momentum distribution to the momentum magnitude becomes smaller, producing more distinct features, and (2) Coincidence events with more fragments contain richer structural information.

1. *Experimental data*

In Supplementary Figure 13, we evaluate the data reduction across three representative fragmentation channels. The Newton plots (a–c), UMAP embeddings (d–f), and distributions of angle between the two chlorine fragments (g–i) all show that fragmentation channels with higher total final-state charges yield clearer separation between geometries.

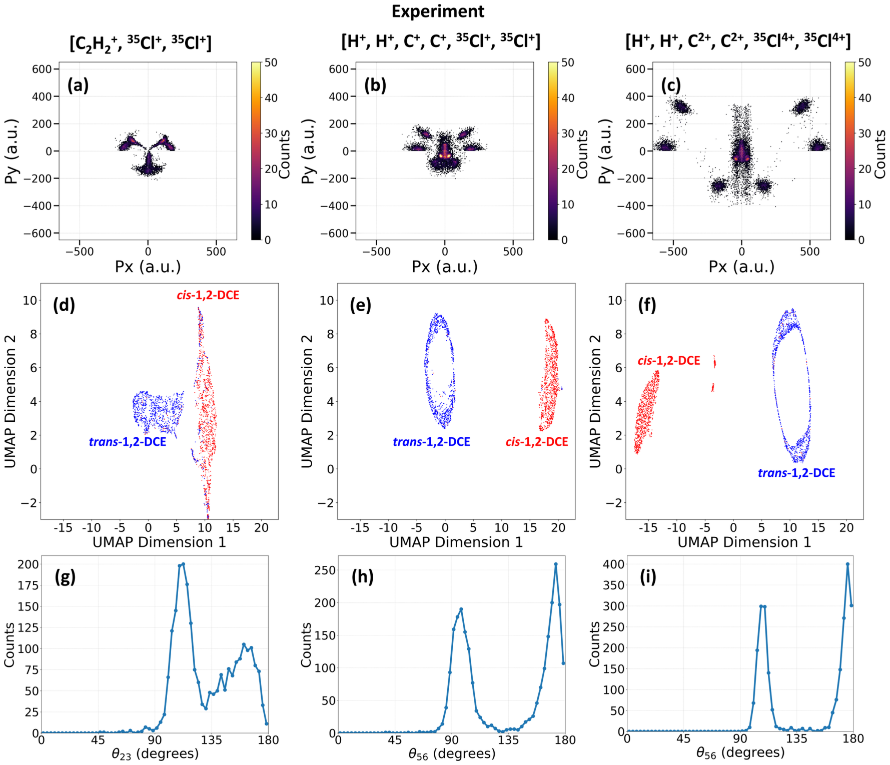

Supplementary Figure 13. **Reduction of experimental data for three fragmentation channels.** (a-c) Newton plots in the molecular plane the three channels: (a) three-body, total charge 3+ ($C_2H_2^+, {}^{35}Cl^+, {}^{35}Cl^+$); (b) six-body, total charge 6+ ($H^+, H^+, C^+, C^+, {}^{35}Cl^+, {}^{35}Cl^+$); (c) six-body, total charge 14+ ($H^+, H^+, C^{2+}, C^{2+}, {}^{35}Cl^{4+}, {}^{35}Cl^{4+}$). (d-f) Two-dimensional Uniform Manifold Approximation and Projection (UMAP) embeddings corresponding to (a–c). (g–i) Distributions of the angle between the two chlorine ions for (a–c). The number of events is fixed across channels, and axis limits are matched for all Newton plots and UMAP panels to enable direct comparison. In UMAP embeddings, points are colored by their true geometry labels.

2. *Simulated data*

In this section, we systematically broadened the initial real-space distributions and increased the kinetic energy for *twisted*-1,2-DCE and 1,1-DCE in the simulation. Across conditions, higher total final-state charge yields more robust

separations of geometries in the UMAP embeddings: clusters in the +14 channel remain distinct as noise or energy grows, whereas the +6 channel progressively overlaps. In all plots, points are colored by their true geometry labels.

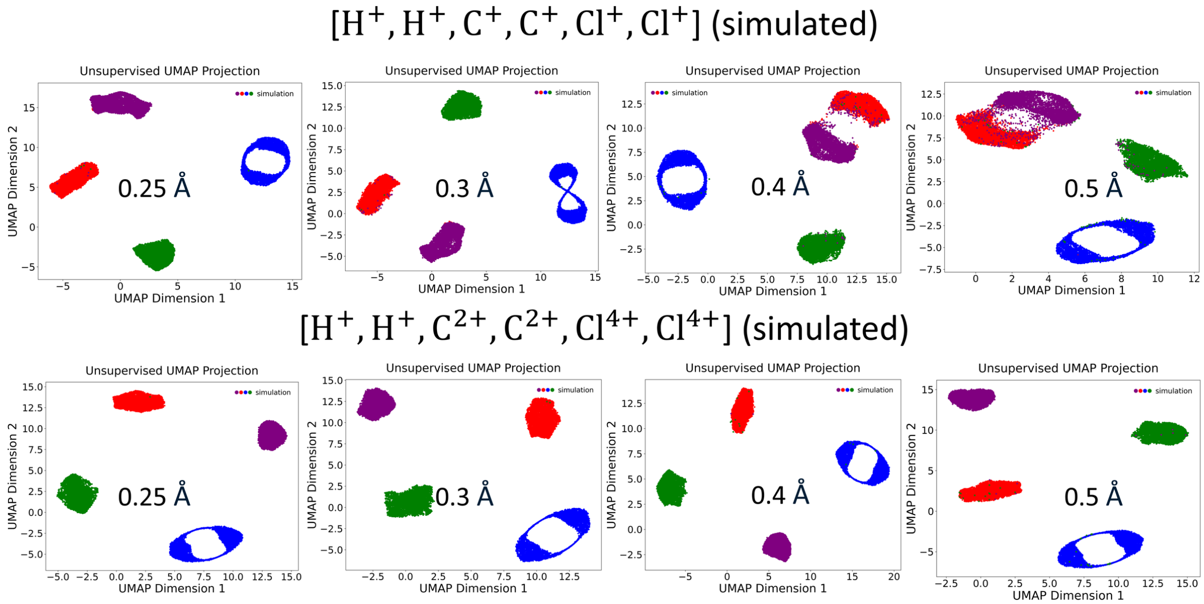

Supplementary Figure 14. **Comparison between two fragmentation channels with a total final charge of +6 and +14.** Simulation parameters for *cis* and *trans* were fixed at 0.25 Å and 0.5 eV. For *twisted*-1,2-DCE and 1,1-DCE, the kinetic energy was set to 0.5 eV, with spatial deviations increasing from 0.25 Å to 0.5 Å (left to right). The +6 channel (top) starts to show overlap between *cis*-1,2-DCE and 1,1-DCE in the last two columns, while the +14 (bottom) channel maintains clear separation throughout. The events are colored by their true labels.

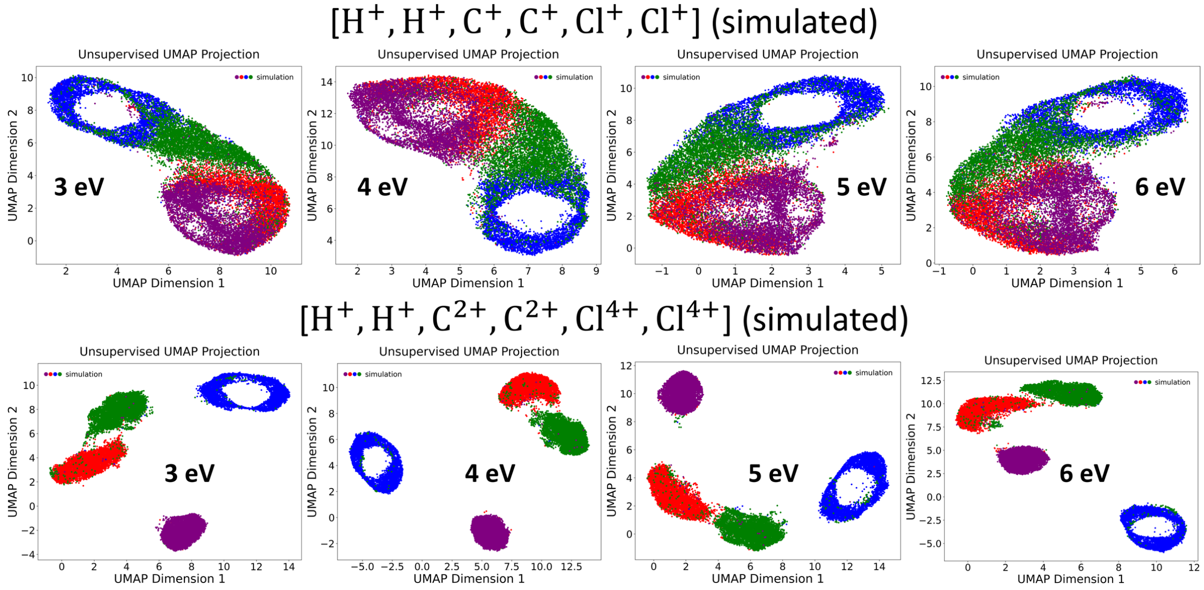

Supplementary Figure 15. **Comparison between the +6 and +14 channels with varying the kinetic energy from 3 eV to 6 eV.** For all geometries, the spatial deviation was kept at 0.5 Å while the kinetic energy was varied from 3 eV to 6 eV (left to right). The +6 channel (top) exhibits substantial overlap between geometries, whereas the +14 channel (bottom) shows consistently well-separated clusters. In all simulations, points are colored by their true geometry labels.

E. Supporting figures for Random Forest Classification analysis

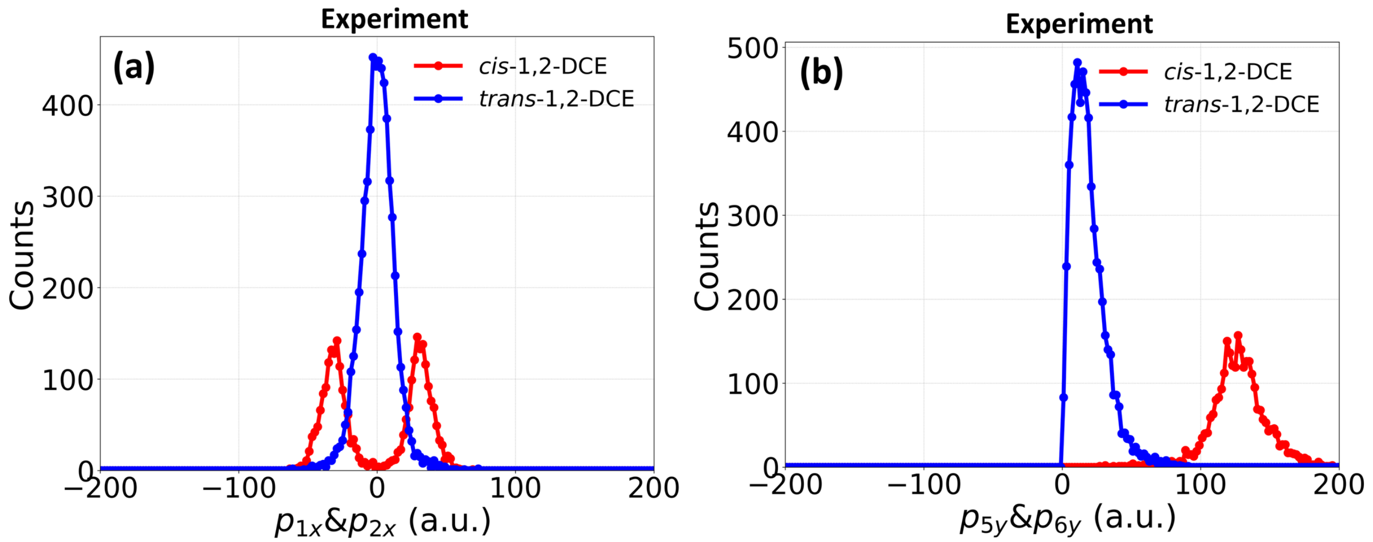

Supplementary Figure 16. **Momentum distributions of the chlorine and proton fragments in the *cis* and *trans*-1,2-DCE isomers.** (a) Horizontal momentum component of protons (p_{1x} and p_{2x}). (b) Vertical momentum component of chlorine ions (p_{5y} and p_{6y}). The separation of the distributions for *cis* and *trans* reflects the structural differences between the isomers and demonstrates that these momentum components are good discriminators for the two isomers, as quantified by our analysis using the Random Forest classifier model.

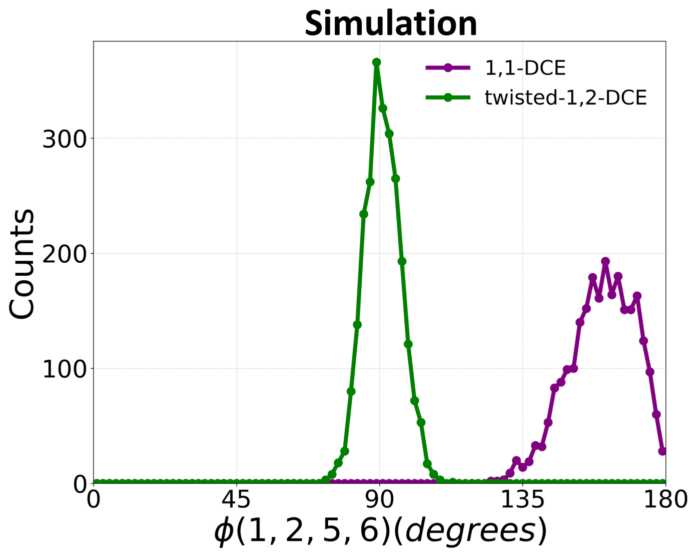

Supplementary Figure 17. **Angle between two planes formed by the (H^+, H^+) and (Cl^+, Cl^+) momentum vectors.** ϕ_{1256} angle serves as a key distinguishing feature between *twisted*-1,2-DCE and 1,1-DCE, showing two well-separated distributions.

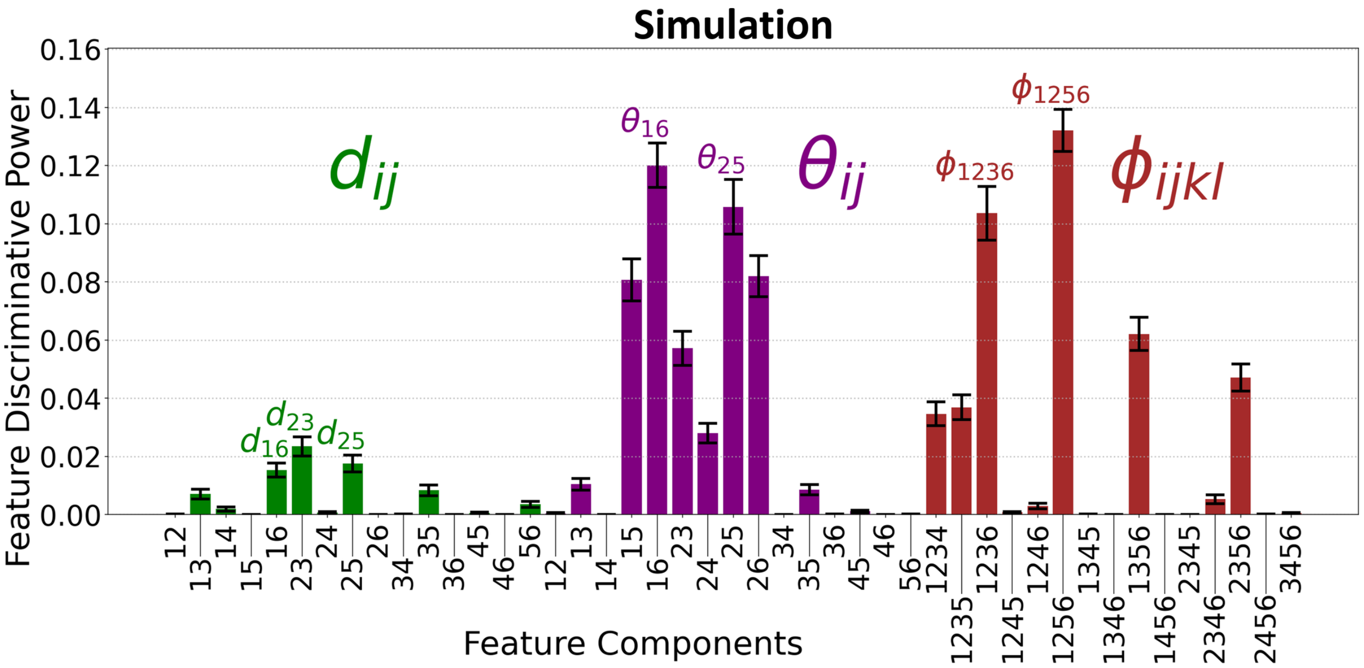

Supplementary Figure 18. **Discriminative power analysis using a Random Forest classifier model applied to distinguish between the *twisted-1,2*-DCE and *1,1*-DCE isomers.** The bar heights indicate the relative discriminative power of different features. The strongest discriminator in this case is ϕ_{1256} — the angle between two planes formed by protons and chlorine ions. Error bars show the standard error of feature importance over 100 Random Forest fits with different random seeds.

Supplementary references

[1] P. Walter, M. Holmes, R. Obaid, L. Amores, X. Cheng, J. P. Cryan, J. M. Glownia, X. Li, M.-F. Lin, M. L. Ng, J. Robinson, N. Shivaram, J. Yin, D. Fritz, J. James, J.-C. Castagna, and T. Osipov, The DREAM Endstation at the Linac Coherent Light Source, Applied Sciences **12**, 10534 (2022).

[2] B. Markovic, C. Bakalis, G. Blaj, X. Defay, D. Doering, A. Gupta, J. Hasi, C. Kenney, P. King, A. Pena-Perez, B. Reese, L. Rota, L. Ruckman, J. Segal, T. Driver, J. Cryan, C. Hansson, and A. Dragone, SparkPix-T: Spatial and Time Resolving Front-End ASIC with MHz-Rate Information Extraction for Momentum Spectroscopy at LCLS-II, in *2023 IEEE Nuclear Science Symposium, Medical Imaging Conference and International Symposium on Room-Temperature Semiconductor Detectors (NSS MIC RTSD)* (2023) pp. 1–1, iSSN: 2577-0829.

[3] X. Li, R. Boll, P. Vindel-Zandbergen, J. González-Vázquez, D. E. Rivas, S. Bhattacharyya, K. Borne, K. Chen, A. De Fanis, B. Erk, R. Forbes, A. E. Green, M. Ilchen, B. Kaderiya, E. Kukk, H. V. S. Lam, T. Mazza, T. Mullins, B. Senfftleben, F. Trinter, S. Usenko, A. S. Venkatachalam, E. Wang, J. P. Cryan, M. Meyer, T. Jahnke, P. J. Ho, D. Rolles, and A. Rudenko, Imaging a light-induced molecular elimination reaction with an X-ray free-electron laser, Nat Commun **16**, 7006 (2025).

[4] B. Richard, R. Boll, S. Banerjee, J. M. Schäfer, Z. Jurek, G. Kastirke, K. Fehre, M. S. Schöffler, N. Anders, T. M. Baumann, S. Eckart, B. Erk, A. De Fanis, R. Dörner, S. Grundmann, P. Grychtol, M. Hofmann, M. Ilchen, M. Kircher, K. Kubicek, M. Kunitski, X. Li, T. Mazza, S. Meister, N. Melzer, J. Montano, V. Music, Y. Ovcharenko, C. Passow, A. Pier, N. Rennhack, J. Rist, D. E. Rivas, D. Rolles, I. Schlichting, L. P. H. Schmidt, P. Schmidt, D. Trabert, F. Trinter, R. Wagner, P. Walter, P. Ziolkowski, A. Rudenko, M. Meyer, R. Santra, L. Inhester, and T. Jahnke, Imaging collective quantum fluctuations of the structure of a complex molecule, Science **389**, 650 (2025).

[5] L. van der Maaten and G. Hinton, Visualizing high-dimensional data using t-SNE, Journal of Machine Learning Research **9**, 2579 (2008).

[6] L. McInnes, J. Healy, and J. Melville, UMAP: Uniform Manifold Approximation and Projection for Dimension Reduction (2018), arXiv:1802.03426 [stat.ML].

[7] P. J. Rousseeuw, Silhouettes: A graphical aid to the interpretation and validation of cluster analysis, Journal of Computational and Applied Mathematics **20**, 53 (1987).

[8] D. L. Davies and D. W. Bouldin, A Cluster Separation Measure, IEEE Transactions on Pattern Analysis and Machine Intelligence **PAMI-1**, 224 (1979).

[9] T. André, I. Dawod, S. Cardoch, E. De Santis, N. Tîmneanu, and C. Caleman, Protein structure classification based on x-ray-laser-induced coulomb explosion, Phys. Rev. Lett. **134**, 128403 (2025).

[10] M. W. Dorrity, L. M. Saunders, C. Queitsch, S. Fields, and C. Trapnell, Dimensionality reduction by UMAP to visualize physical and genetic interactions, Nat Commun **11**, 1537 (2020).
